# Microstructure effects on the phase transition behavior of a prototypical quantum material

**DOI:** 10.1038/s41598-022-13872-0

**Published:** 2022-06-21

**Authors:** Jan O. Schunck, Florian Döring, Benedikt Rösner, Jens Buck, Robin Y. Engel, Piter S. Miedema, Sanjoy K. Mahatha, Moritz Hoesch, Adrian Petraru, Hermann Kohlstedt, Christian Schüßler-Langeheine, Kai Rossnagel, Christian David, Martin Beye

**Affiliations:** 1 Deutsches Elektronen-Synchrotron DESY, Notkestraße 85, 22607 Hamburg, Germany; 2 Physics Department, Universität Hamburg, Luruper Chaussee 149, 22761 Hamburg, Germany; 3 Paul Scherrer Institut (PSI), Forschungsstraße 111, 5232 Villigen, Switzerland; 4 Institut für Experimentelle und Angewandte Physik, Kiel University, Olshausenstraße 40, 24098 Kiel, Germany; 5 Ruprecht-Haensel-Labor, Deutsches Elektronen-Synchrotron DESY, Notkestraße 85, 22607 Hamburg, Germany; 6 Faculty of Engineering, Chair of Nanoelectronics, Kiel University, Kaiserstraße 2, 24143 Kiel, Germany; 7 Helmholtz-Zentrum Berlin für Materialien und Energie, Albert-Einstein-Straße 15, 12489 Berlin, Germany; 8 Present Address: School of Physics and Materials Science, Thapar Institute of Engineering and Technology, Patiala, 147004 India

**Keywords:** Electronic properties and materials, Phase transitions and critical phenomena, Surfaces, interfaces and thin films, Electronic and spintronic devices, Electronic devices

## Abstract

Materials with insulator-metal transitions promise advanced functionalities for future information technology. Patterning on the microscale is key for miniaturized functional devices, but material properties may vary spatially across microstructures. Characterization of these miniaturized devices requires electronic structure probes with sufficient spatial resolution to understand the influence of structure size and shape on functional properties. The present study demonstrates the use of imaging soft X-ray absorption spectroscopy with a spatial resolution better than 2 $\upmu$m to study the insulator-metal transition in vanadium dioxide thin-film microstructures. This novel technique reveals that the transition temperature for the conversion from insulating to metallic vanadium dioxide is lowered by 1.2 K ± 0.4 K close to the structure edges compared to the center. Facilitated strain release during the phase transition is discussed as origin of the observed behavior. The experimental approach enables a detailed understanding of how the electronic properties of quantum materials depend on their patterning at the micrometer scale.

## Introduction

Quantum materials are characterized by exotic physical properties arising from correlations and inter-dependencies of electron spin, charge and orbital degrees of freedom, as well as the lattice. The interaction of these subsystems results in intriguing functional properties like high-temperature superconductivity or insulator-metal transitions^1–3^. Current and future devices require patterning of such materials on the micro- or nanometer scale. Designing such devices requires an understanding of how bulk material properties are preserved or altered across these miniaturized structures since, e.g., the relatively enlarged surface areas can facilitate volume strain relaxation. Strain effects become even more important when the functionality of the device is derived from solid-solid phase transitions, like insulator-metal transitions, during which changes of lattice parameters occur. For example, strain in thin-films, mediated via the substrate, has been observed to significantly affect the phase transition temperature^4,5^.

In quantum materials research, vanadium dioxide (VO$_2$) is a prototypical insulator-to-metal transition (IMT) compound with a transition temperature $T_t$ of approximately 340 K^6^. The proximity of this solid-solid phase transition to room temperature along with a change in resistivity of up to five orders of magnitude makes VO$_2$ a particularly interesting compound for applications (see^7^ and references therein), e.g., as a phase change material in optical waveguide switches^8^, for optical^9^ or memristive^10,11^ devices, thermal sensors or thermochromic coatings^12^ on multifunctional windows^13^, as well as neuromorphic circuits^14^.

On the microscopic level, the IMT in VO$_2$ is characterized by a combined change of crystallographic and electronic structure. The metallic high-temperature phase of VO$_2$ has a rutile crystal structure. According to the Goodenough model^15^, the crystal symmetry decreases upon cooling of the material when two adjacent vanadium atoms move closer toward each other, forming a monoclinic lattice. The valence and conduction bands in VO$_2$ are formed by hybridization of vanadium 3*d* and oxygen 2*p* orbitals. In the metallic phase, the conduction band consists of directional $d_{||}$ and unidirectional $\pi ^*$ bands^16^. Due to the dimerization during the formation of the insulating phase, the $d_{||}$ band splits into bonding ($d_{||}$) and antibonding ($d_{||}^*$) contributions, and the $\pi ^*$ band is lifted in energy. These electronic structure effects jointly cause the opening of a band gap around the Fermi level.

X-ray absorption spectroscopy (XAS) probes the unoccupied density of states. The shift of unoccupied bands during the IMT thus makes XAS an ideal and routinely used tool to follow the IMT in VO$_2$^16–19^. In particular, XAS at the oxygen *K*-edge probes the $1s-2p$ transition in oxygen, making it most sensitive to the nature of the phase of VO$_2$^16^. We note that a different monoclinic, insulating phase (termed $M_2$), has been proposed for strained or Cr-doped VO$_2$ samples^20–22^. To our knowledge, no X-ray absorption spectra of this phase are known. Thus, our analysis omits a potential influence of this phase.

In VO$_2$ thin-films, it has been observed that the IMT progresses via domain formation in the vicinity of the transition temperature $T_t$^23–26^. Across a large temperature range of up to 50 K around $T_t$, insulating and metallic domains coexist^25^. Upon heating, metallic islands form, grow and build a domain network within the insulating phase. The size of these domains typically is on the sub-micron scale^24,25,27^. Therefore, structuring VO$_2$ thin-films on the micrometer length scale, as required for devices, naturally influences the domain network. In literature, different forms of micro- and nano-structuring have been shown to alter $T_t$ by approximately 5 K to 15 K^28–32^. However, the question whether this happens homogeneously across the structures has not yet been addressed.

## Results

**Figure 1:**
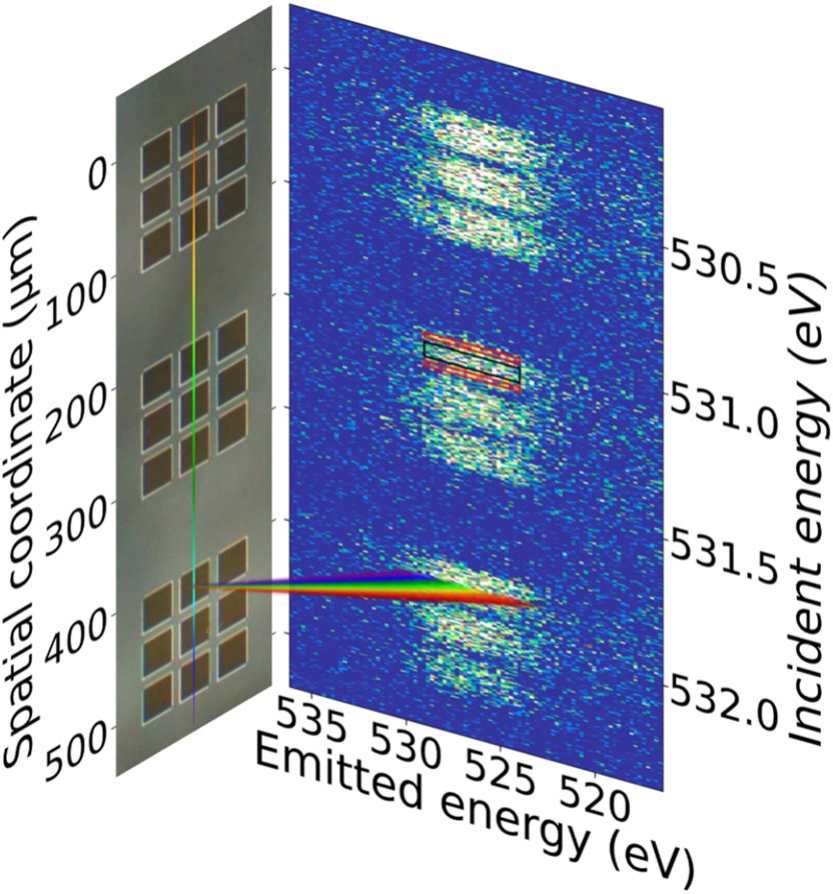
Imaging of the microstructured VO$_2$ thin film sample. Left: Optical microscopy image of the microstructures. The vertical rainbow-colored line illustrates the illuminating X-ray line focus with varying incident photon energy. Right: Background subtracted detector image of oxygen fluorescence from the microsquares with spatial resolution along the vertical axis. Three rectangles highlight the regions of interest for edge (red) and center regions (black). Note that both panels are scaled to the same vertical spatial axis. Due to the space-energy coupling along the line focus (see text), the incident energy also varies along the vertical dimension.

In this study, we use imaging soft X-ray absorption spectroscopy with a spatial resolution of down to 1.8 $\upmu$m to investigate the thermally driven IMT in supported thin-film VO$_2$ squares of 30 $\upmu$m $\times$ 30 $\upmu$m size (see Fig. 1). In brief, the combination of imaging and spectroscopy is realized with an off-axis Fresnel zone plate between sample and two-dimensional CCD detector. The zone plate is used to image and disperse X-ray emission simultaneously onto orthogonal dimensions of the detector^33,34^. As a measure for the X-ray absorption, we observe oxygen *K*-edge fluorescence within an emission energy window of 526.5 eV ± 2.5 eV. A more detailed characterization of the experimental setup can be found in a previous publication^35^.

With the beamline optics and a linear illumination zone plate, incident X-ray radiation is focused onto the sample as a vertical line of approximately 970 $\upmu$m length and sub-micron width. Due to dispersion by the beamline monochromator, the photon energy along the line focus changes linearly by approximately ± 1.8 eV, centered around the monochromator energy.

The sample is a bulk-supported 50 nm thin microstructured VO$_2$ film (see Experimental section and the reference^36^). The structures are squares with an edge length of 30 $\upmu$m, arranged in groups of three by three (see Fig. 1). The X-ray line focus extends across several groups of VO$_2$ microsquares. Figure 1 shows a typical, background subtracted detector image. The horizontal is the dispersive direction of the analyzer zone plate and thus shows the fluorescence on an emission energy axis. The vertical is the imaging direction of the analyzer zone plate and thus encodes vertical position on the sample surface as well as the systematic incident energy variation. The signal of three groups, each containing three microsquares, can be clearly recognized.

**Figure 2:**
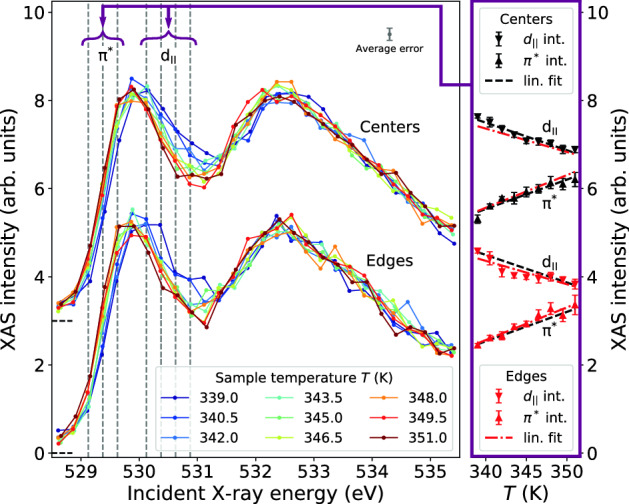
Left panel: Partial fluorescence yield oxygen *K*-edge X-ray absorption spectra from center (top) and edge (bottom) regions averaged over five VO$_2$ microsquares for temperatures ranging from 339 to 351 K. The average standard deviation for a single data point is displayed in the upper right corner. Vertical grey dashed lines indicate the energies which were chosen for extraction of the metallic fraction (see main text and Fig. 3). Right panel: Mean intensities in the $\pi ^*$ and $d_{||}$ spectral regions show the IMT progression. Within each spectral region, black and red triangles respectively show the average intensity for the spectra from centers and edges. Additionally, linear fits to the intensity trends of the centers and edges are shown as guide to the eye using black, dashed and red, dash-dotted lines, respectively. Error bars show the one-sigma standard deviation of intensities of the five VO$_2$ microsquares in each energy region. In both panels, data for the center regions have been shifted up by three units for display.

In order to investigate to which extent patterned thin-films exhibit inhomogeneous phase transition behavior, temperature series of X-ray absorption spectra are recorded by scanning the incoming photon energy across the oxygen *K*-edge from 527 to 536 eV for nine different temperatures from 339 to 351 K (i.e., in steps of 1.5 K). In the imaging direction, the signal from edge and center regions of the microsquares is analyzed separately in regions of interest (ROIs) of 4.5 $\upmu$m and 12.3 $\upmu$m height close to the edges of the squares and in the square centers, respectively. Between the center region and each edge region, a spacing of 4.4 $\upmu$m was excluded from the analysis. Exemplary ROIs for edges and centers are shown for one square in Fig. 1. Data presented hereafter is derived from the evaluation of the second to sixth square (counted from top to bottom) with corresponding ROIs. The other squares moved out of the X-ray focus during the experiment when the sample holder expanded due to the increase in temperature. For the evaluated squares, the signal within each ROI is integrated and plotted against the incident energy (corrected for the monochromator dispersion along the line focus) resulting in the partial fluorescence yield X-ray absorption spectra shown in Fig. 2.

The spatially resolved X-ray absorption spectra, extracted from edge and center regions of the VO$_2$ microsquares for different temperatures around $T_t$ show very good agreement with spectra from literature^19,37–39^. In the case of center regions, this is true for all spectral features and the corresponding temperatures, while in the case of edge regions, deviations are observed for the temperature dependence. Upon heating, the largest changes in the oxygen *K*-edge absorption spectra induced by the IMT are found in two spectral regions: intensity of the $d_{||}$ shoulder at 530.3 eV is decreased and the leading edge of the $\pi ^*$ peak between 529 and 530 eV is red-shifted. Systematic changes continue towards higher temperatures beyond the presently studied temperature range. Despite statistical uncertainties, we already observe that these spectral changes upon heating occur earlier in the regions close to the edge of the microsquares as compared to regions in the center. In the right panel of Fig. 2, we display further evidence for this observation, gaining statistical significance by averaging in the relevant regions: The average spectral intensity increase in the $\pi ^*$ region and the intensity decrease in the $d_{||}$ region with increasing temperature are both more advanced for the edges (red) in comparison to the centers (black). As a guide to the eye, linear fits of the edge evolutions (red, dash-dotted line) are shown alongside the data and linear fits of the center evolutions (black dashed lines) and vice versa.

## Analysis

With the given spatial resolution of 1.8 $\upmu$m, we can assume that each spectrum contains contributions from metallic and insulating domains. In the case of extended VO$_2$ thin-film layers, the domain size has been shown to be hundreds of nanometers in size^25,27^, and we can expect a similar domain size also in the edge regions of the squares. When the VO$_2$ sample is heated across the IMT, the measured signal is thus an average of the relative contributions from the continuously growing, respectively shrinking, metallic and insulating domains. In consequence, spectral changes observed in XAS do not resolve an abrupt 1st order IMT but are continuous with temperature. The spectra provide a measure for the fraction of the sample which has undergone the IMT, which we refer to as metallic fraction. For a quantification of the metallic fraction, we refer to recent X-ray absorption measurements at the oxygen *K*-edge, where quasi-linear IMT-induced changes over a temperature range of approximately 40 K have been reported^38^. The low-temperature onset was observed at 335 K in our setup, which deviates slightly from the reported one^38^. We attribute this to different positions of temperature sensor and sample. Accordingly, we assume the metallic fraction to increase linearly from 0 at 335 K to 1 at 375 K.

Analysing the characteristic changes in the $\pi ^*$ and $d_{||}$ spectral regions then allows us to directly extract the fraction of metallic VO$_2$ in the sample. We assume that the spectra from the center regions are showing the same temperature dependence as the spectra from unstructured films. We further assume that both the metallic fraction as well as the spectral changes are linear in temperature. Thus, we can extract the metallic fraction directly from the spectra and apply the same analysis procedure also to the spectra from the edge regions to quantify the deviations.

In detail, we connect spectra to the fraction of metallic VO$_2$ as follows: Within the energy region with the most prominent spectral changes around the $\pi ^*$-peak from 529 eV to 531 eV, we select seven intensity data points $I(E_i, T)$ from the center region measurement marked by gray dashed lines in Fig. 2. The intensity-temperature relations of the center region data points are mapped to intensity-metallic fraction relations using the assumed linear dependence of temperature and metallic fraction for unstructured thin films (see above). These seven relations are linearly fitted and yield a set of parameters which allows to determine the metallic fraction of any other spectrum evaluated from the measured intensities at these energy points. The obtained seven values are then averaged. We note that the region around the maximum of the $\pi ^*$-peak at 529.9 eV shows no temperature dependence and is thus not sensitive to the changes in metallic fraction. This region was excluded from the analysis.

## Discussion

**Figure 3:**
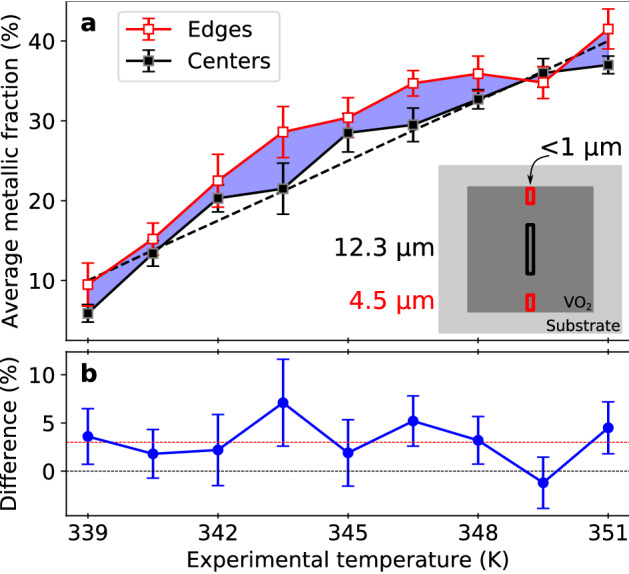
Comparison of average metallic fraction of center (black filled squares) and edge (red empty squares) regions of VO$_2$ microsquares for experimental temperatures around the transition temperature $T_t$. Each data point in panel (**a**) is the average of seven metallic fractions, determined from different energies in the X-ray absorption spectrum (see Fig. 2). Error bars represent the standard deviation of those seven values. The dashed black line is a guide to the eye and represents a perfectly linear increase of the metallic fraction with experimental temperature. The inset visualizes the regions of interest (ROIs) on a VO$_2$ square from which the signal for respective data color was extracted. In horizontal direction the dimensions of the ROIs are determined by the width of the vertical X-ray line focus. In the vertical, the ROI dimensions are chosen. The blue shaded area shows the difference in metallic fractions of center and edge regions, which is additionally plotted in panel (**b**). The black and red horizontal line in **b** are a guide to the eye at difference values of 0% and 3%.

The resulting average metallic fractions for all measured spectra are shown in Fig. 3A. The black and red data points in Fig. 3A show the average metallic fraction for the temperature series of the microsquare center and edge regions. Within the experimental uncertainty, the fraction of metallic domains is consistently larger at the edge regions of the VO$_2$ squares in comparison to the center regions for the temperature range studied in this work. The average metallic fraction at the edge regions also shows the same curved deviation from linearity as the center regions, but with an offset of $3 \pm 1$ percentage points on average (Fig. 3B). A change of metallic fraction of $3 \pm 1$ percentage points translates to a temperature difference of 1.2 K ± 0.4 K. Upon heating, the edge regions thus reach the same state (metallic fraction) in the the phase transition as the centers at a 1.2 K ± 0.4 K lower temperature. This value is independent of the chosen scaling of the metallic fraction. We emphasize that both center and edge regions are measured at the same time under the same sample conditions. Furthermore, we found no systematic deviation between different squares.

Some previous reports have examined the effects of structure size on the transition temperature of VO$_2$ micro- and nanostructures and reported two opposing trends: On the one hand, studies of size effects in VO$_2$ nanoprecipitates, prepared in a SiO$_2$ substrate by ion implantation and subsequent high-temperature annealing^29^, as well as of VO$_2$ nanoparticles capped with gold^40^, report a widening of the IMT hysteresis with decreasing particle size, i.e., smaller particles with larger relative surface area show on average higher transition temperatures $T_t$ upon heating and lower $T_t$ upon cooling. These results are at odds with our data, where edge regions with increased surface area show a lower transition temperature upon heating.

On the other hand, different reports on VO$_2$ nano- and microstructures are consistent with our findings: For VO$_2$ nanowires on a SiO$_2$ surface, the transition temperature is lowered upon heating for thinner nanowires^31^, i.e., for larger relative surface area. A similar trend is observed on rod-like VO$_2$ nanostructures of subsequently smaller sizes^30^, for nanowires between 3.5 $\upmu$m and 100 nm width on a Si wafer^28^ and for films with varying grain size^32^. This effect is commonly attributed to an enriched defect (i.e., nucleation site) density at the surface, which, for smaller structures, is larger with respect to the bulk.

The contradicting results^29,40^ (i.e., more surface area leading to an increased transition temperature upon heating) likely originate from the surface treatment of the VO$_2$ structures, as they were either implanted in a SiO$_2$ matrix in the first case or capped with gold in the second case. As a result, surface bonds could be saturated and substantially fewer defects were potentially formed at the surface of the VO$_2$ nanostructures.

The VO$_2$ microstructures which we report on here are also expected to possess a higher defect density, such as oxygen vacancies^41–43^, at the edge regions of the squares, arising for example from the etching process during structuring of the sample. However, we do not consider enriched nucleation site density at lateral edges to play a substantial role during the phase transition of our microsquares. Firstly, by far the biggest contribution to the outer boundaries of these structures comes from the film top surface which is parallel to the substrate. The lateral edge surfaces increase the total surface area of the measured edge region only by less than 1%. Secondly, we assume that the higher defect density influences only the first few layers of the crystal. The ROI of the edges in which we observe the IMT at lower temperatures, however, was set to measure 4.5 $\upmu$m in width, which corresponds to around 10,000 layers. Thus, by averaging within this ROI, higher defect site density at the lateral edge regions becomes negligible in comparison to the volume effects. This is further supported by the observation that, within the experimental sensitivity, the slope of the metallic fraction over temperature (Fig. 3A) is unchanged between center and edge regions. On the contrary, a change in the local distribution of nucleation centers would be expected to promote a faster completion of the IMT (i.e., a steeper slope in the metallic fraction-temperature diagram), rather than the observed shift of the overall phase transition temperature.

Thus, we assume a different cause for the different transition temperatures of edge and center regions: Strain, induced by a change in crystal lattice constants during the phase transition, can add up across micrometers^44,45^. In our experiment, strain relaxes more easily at the edge regions in comparison to the more-confined center regions. Strain changes the potential energy landscape and thus the energy balance of the subsystems driving the IMT, which apparently leads to an earlier onset of the IMT with a similar slope as a function of temperature. Thus, we assess that an eased strain relaxation at the edge regions could lead to the overall lowering of the transition temperature by 1.2 K ± 0.4 K on average. Moreover, we assume that the effect of facilitated strain release on the transition temperature is much larger at the microsquare edges and decreases toward the center.

## Conclusions

In summary, we have utilized X-ray imaging spectroscopy with micrometer resolution for the characterization of the insulator-metal transition in 30 $\upmu$m $\times$ 30 $\upmu$m sized VO$_2$ thin film structures. Quantitative comparison of the phase transition-induced shift in the X-ray absorption spectra at the oxygen *K*-edge revealed that the transition temperature upon heating was lowered by 1.2 K ± 0.4 K at the edge regions of microstructures in comparison to center regions. Our findings suggest an eased release of strain which is built up during the crystallographic phase transition. These results demonstrate how shape and pattern influence the functional properties of materials on the microscale. Systematic studies of phase transitions of microscopically patterned quantum material systems will be indispensable in order to be able to accurately tailor functional devices. Future studies with improved spatial resolution will furthermore enable a more detailed analysis of the length scales involved in the build-up and release of strain during solid-solid phase transitions.

## Methods

### Imaging soft X-ray absorption spectroscopy measurements

Spatially resolved X-ray absorption partial fluorescence yield measurements were performed with the MUSIX endstation^46^ located at the soft X-ray beamline P04^47^ of the synchrotron storage ring PETRA III at DESY in Hamburg. Characteristics of the setup are discussed in the reference^35^. The setup makes use of the entire bandwidth that is transmitted through the beamline monochromator. A 1200 lines  mm$^{-1}$ grating was used and the exit slit was opened as far as possible (2.9 mm). At 530 eV photon energy, the monochromator dispersion was approximately 1.3 eV  mm$^{-1}$, leading to the transmitted bandwidth of ± 1.8 eV, mentioned in the main text. The refocusing mirrors downstream of the monochromator were used to focus the dispersed beam only in the horizontal direction. The vertical focal point of the refocusing mirrors was several meters downstream of the sample. The vertical line focus was instead created with a linear illumination zone plate to create a narrower line focus than possible with the refocusing mirrors. The illumination zone plate was located about 15 cm upstream of the sample. As a result, a sub-micron wide vertical focus line of approximately 970 $\upmu$m length was obtained. Along the direction of the vertical line focus, the sample was imaged with an off-axis zone plate, which additionally dispersed X-rays emitted from the sample in horizontal direction. Object and image distances were 15 cm and 172.5 cm respectively, resulting in a sample magnification of 11.5. Movement of the off-axis zone plate along the sample-detector axis allows focusing different energies of the emission spectrum onto the CCD detector. The detector was a CCD camera (Andor iKon) with a pixel size of 13 $\upmu$m. An aluminium foil was used to protect the detector from visible light.

For the X-ray absorption measurements, the central photon energy was scanned around the oxygen *K*-edge from 527 to 536 eV. Because of the bandwidth transmitted through the open exit slit, every point on the sample is illuminated by photon energies between 527 eV + 1.8 eV and 536 eV − 1.8 eV. The photon energy was scanned in steps of 0.25 eV with 60 s integration time per step, resulting in less than 40 min acquisition time per temperature. Between absorption scans, the sample temperature was increased from 339 to 351 K in steps of 1.5 K. After each temperature increase, the sample was given 40 minutes of equilibration time.

### Sample fabrication

The 50 nm thin vanadium dioxide film was deposited on a commercial Al$_2$O$_3$ (0001) single crystal substrate (supplied by Shinkosha, Japan) by pulsed laser deposition, using a KrF excimer laser of 248 nm wavelength and a commercial sintered ceramic V$_2$O$_5$ target (supplied by Evochem, Germany). The laser fluence during deposition was 2 J cm$^{-2}$. The structures were created using optical lithography and ion beam etching. The etching process was monitored using a secondary ion mass spectrometer. Etching was stopped after the part of the VO$_2$ film that was not protected by the photo resist was removed entirely.

## Data Availability

The datasets generated during and/or analysed during the current study are available from the corresponding author on reasonable request.
